# Demographic and risk group heterogeneity across the UNAIDS 90-90-90 targets: a systematic review and meta-analysis protocol

**DOI:** 10.1186/s13643-019-1024-6

**Published:** 2019-05-06

**Authors:** Dylan Green, Brenda Kharono, Diana M. Tordoff, Adam Akullian, Anna Bershteyn, Michelle Morrison, Geoff Garnett, Ann Duerr, Paul Drain

**Affiliations:** 1 Departments of Epidemiology and Global Health, University of Washington, 1959 NE Pacific St, Seattle, 98195 Washington USA; 2 Strategic Analysis, Research & Training (START) Center, University of Washington, 1510 San Juan Rd NE, 3rd floor, Seattle, Washington 98195 USA; 3 Institute for Disease Modeling, 3150 139th Ave SE, Building IV, Bellevue, 98005 Washington USA; 4 Bill and Melinda Gates Foundation, 500 5th Ave N, Seattle, 98109 Washington USA; 5 HIV Vaccine Trials Network, Vaccine and Infectious Disease and Public Health Science Divisions, Fred Hutchinson Cancer Research Center, 1100 Fairview Ave N, Seattle, 98109 Washington USA; 6 Division of Allergy and Infectious Diseases, Department of Medicine, School of Medicine, University of Washington, 1959 NE Pacific St, Seattle, 98195 Washington USA

**Keywords:** HIV, Care cascade, HIV diagnosis, Antiretroviral therapy, Viral suppression, Virological suppression, 90-90-90, Risk-factors, Systematic review, Meta-analysis, Africa

## Abstract

**Background:**

Despite policies for universal HIV testing and treatment (UTT) regardless of CD4 count, there are still 1.8 million new HIV infections and 1 million AIDS-related deaths annually. The UNAIDS 90-90-90 goals target suppression of HIV viral load in 73% of all HIV-infected people worldwide by 2030. However, achieving these targets may not lead to expected reductions in HIV incidence if the remaining 27% (persons with unsuppressed viral load) are the drivers of HIV transmission through high-risk behaviors. We aim to conduct a systematic review and meta-analysis to understand the demographics, mobility, geographic distribution, and risk profile of adults who are not virologically suppressed in sub-Saharan Africa in the era of UTT.

**Methods:**

We will review the published and grey literature for study sources that contain data on demographic and behavioral strata of virologically suppressed and unsuppressed populations since 2014. We will search PubMed and Embase using four sets of search terms tailored to identify characteristics associated with virological suppression (or lack thereof) and each of the individual 90-90-90 goals. Record screening and data abstraction will be done independently and in duplicate. We will use random effects meta-regression analyses to estimate the distribution of demographic and risk features among groups not virologically suppressed and for each individual 90-90-90 goal.

**Discussion:**

The results of our review will help elucidate factors associated with failure to achieve virological suppression in sub-Saharan Africa, as well as factors associated with failure to achieve each of the 90-90-90 goals. These data will help quantify the population-level effects of current HIV treatment interventions to improve strategies for maximizing virological suppression and ending the HIV epidemic.

**Systematic review registration:**

PROSPERO CRD42018089505.

**Electronic supplementary material:**

The online version of this article (10.1186/s13643-019-1024-6) contains supplementary material, which is available to authorized users.

## Background

In 2016, there were approximately 36.7 million people living with HIV (PLHIV), 1.8 million new HIV infections, and 1 million AIDS-related deaths [1]. In 2014, the Joint United Nations Programme on HIV/AIDS (UNAIDS) established the 90-90-90 goals for the HIV care cascade. The HIV care cascade and these goals aim for 90% of PLHIV to know their status, of whom 90% link to and initiate antiretroviral treatment (ART) and of whom 90% achieve virological suppression by 2020 [2]. UNAIDS modeling projections estimate that new HIV infections will be reduced by up to 90% if the global community achieves the ambitious UNAIDS 90-90-90 goals by 2030 [3].

A successful program for the 90-90-90 goals will achieve virological suppression in 73% (90% × 90% × 90%) of PLHIV, which will leave 27% of PLHIV—approximately 9.9 million people—without achieving virological suppression. Virological suppression occurs when an infected individual’s HIV RNA level drops below a particular threshold and results in improved health outcomes for the individual and reduced risk of onward transmission. If those who do not achieve HIV virological suppression are the drivers of HIV transmission by engaging in high-risk behaviors, then there is a strong risk that achieving the 90-90-90 targets will not lead to ending the HIV epidemic [4]. Therefore, incorporating data on the risk profile of the missing 27% who are not virologically suppressed may help to more accurately predict the impact of achieving the 90-90-90 targets [5]. This will also inform HIV programs to develop strategies, in addition to universal testing and treatment (UTT), that are most beneficial to ending the HIV epidemic [6].

Simulation studies, observational data, and randomized trials have had mixed results in estimating the population benefits of UTT. Mathematical modeling based on results of a cluster-randomized trial of UTT and circumcision in Zambia and South Africa estimated reductions of incidence between 25% and 62% over 10 years [6]. A large population-based cohort in South Africa found a considerable reduction in HIV incidence across communities with high levels of ART coverage [7]. However, completed randomized trials of UTT demonstrated limited population-level effectiveness, which supports the need for more research on the role that groups which are not virologically suppressed play in sustaining high levels of HIV incidence [8]. Empirical evidence is needed to determine the characteristics of people who are not reached through the 90-90-90 strategy, which may inform future strategies to curb onward HIV transmission.

We report herein our protocol for a systematic review and meta-analysis of the demographic, epidemiologic, sexual-risk behavior, and geographic heterogeneity across the HIV care cascade throughout sub-Saharan Africa.

## Aims

The primary aim of this systematic review and meta-analysis is to characterize the HIV transmission potential (defined by demographic and other risk group characteristics) of populations not virologically suppressed in the era of UTT in sub-Saharan Africa. The second aim of this review is to quantify the demographic and risk group heterogeneity at each of the individual 90-90-90 goals—awareness of HIV infection, ART initiation, and HIV virological suppression. We will identify and use subpopulations and risk group strata that have been defined, find comparable strata by which to quantify heterogeneity across the 90-90-90 cascade, and calculate pooled point estimates of the proportion who do not achieve virological suppression within these strata.

## Methods

This systematic review protocol follows the Preferred Reporting Items for Systematic Review and Meta-Analysis Protocols (PRISMA-P) guidelines [9]. The PRISMA-P checklist can be reviewed as Additional file 1. This review protocol is registered on the International Prospective Register of Systematic Reviews database (PROSPERO CRD42018089505) [10].

### Eligibility criteria

#### Study design and settings

We will include all observational cohort studies, case-control studies, cross-sectional studies, HIV surveillance reporting, and randomized control trials that report on one or more elements of the 90-90-90 HIV care cascade. We will include studies that report aggregate estimates or stratum-specific estimates. We will exclude studies that provide modeled estimates of testing, treatment, or virological suppression, systematic reviews and meta-analyses, and data collected exclusively prior to January 2014.

#### Population

We will include any study of adult populations, defined as PLHIV aged 15 years or older, located in sub-Saharan Africa. We will include strata defined in Table 1.

**Table 1:** Demographic and risk group strata of interest

Age	
Sex	
Subnational administrative unit	
Urban, peri-urban, or rural residence	
Occupation	
Active military	
Distance from facility	
Out of pocket payments	
Marital status	
Age at sexual debut	
CD4 count	
Timing of last HIV test	
Education attainment	
Income	
Migratory populations	
Recency of migration	
Mobile populations	
Depression status	
Anxiety status	
Opportunistic infections	
Number of concurrent sexual partners	
Number of previous sexual partners	
Age of sexual partners	
Condom use	
Pregnant women	
Discordant couples	
Injection drug users	
Transactional sex/commercial sex workers	
Gay, bisexual, and other men who have sex with men	
Transgender individuals	

#### Outcomes

The primary outcome is the prevalence of unsuppressed viral load by demographic and risk group, and risk factors associated with being unsuppressed. The labels and definitions of strata used in included studies will be recorded to document the diversity and similarity of strata categorization (e.g., mobile versus migratory populations). The secondary outcomes of interest are defined as the proportion, relative risk, or odds ratio of the following three groups: PLHIV who know their HIV status, PLHIV who know their status and are receiving ART, and PLHIV on ART who are virologically suppressed.

### Information sources

#### Electronic databases

**Fig. 1:**
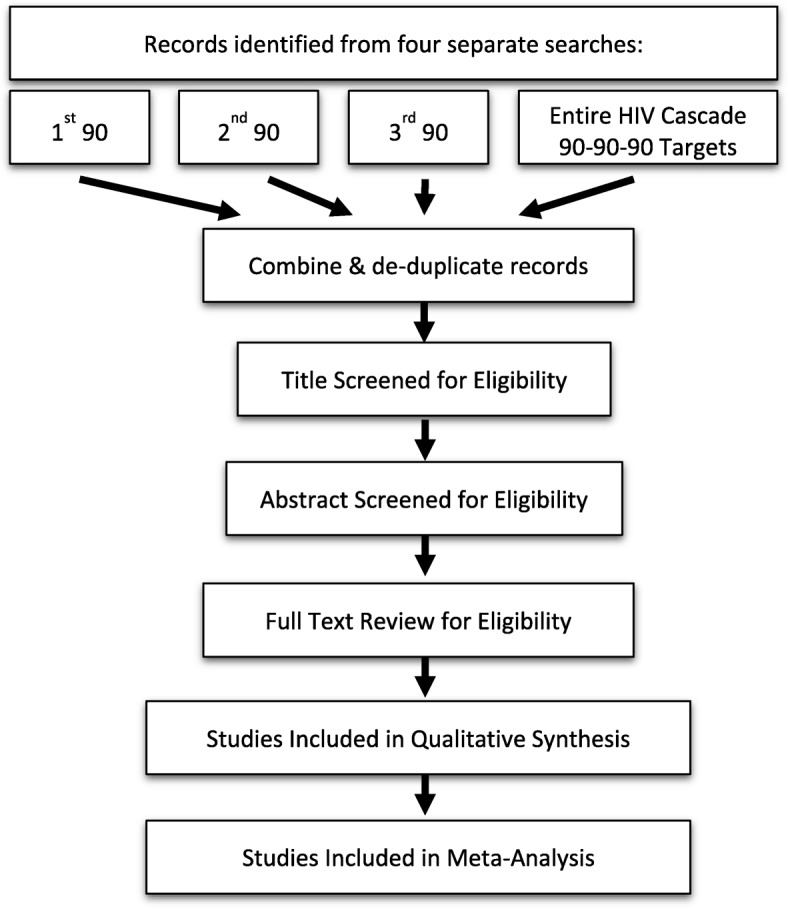
Search strategy

We will conduct four comprehensive literature searches, one for each of the 90-90-90 targets and a fourth search focusing on the entire HIV care cascade (Fig. 1). We will perform searches in PubMed and Embase and restrict the search to articles published in English between January 2014 and March 2018. We will limit our search to articles published after 2014 because the UNAIDS 90-90-90 targets were announced mid-2014 and the World Health Organization (WHO) universal test and treat guidelines were released in 2015 [11].

In addition to searching the published peer-review literature, we will conduct a comprehensive search of websites and databases for publicly available grey literature sources, such as WHO and UNAIDS reports, Ministries of Health websites and national surveillance reports, Médecins Sans Frontières (MSF) reports, the Integrated Bio-Behavioral Surveys (IBBS), U.S. President’s emergency plan for AIDS Relief (PEPFAR) country operational plans, the Analyzing Longitudinal Population-based HIV/AIDS data on Africa (ALPHA) Network, the National Technical Reports Library (NTRL), the Population-based HIV Impact Assessments (PHIA), AIDS Indicator Surveys (AIS), Demographic and Health Surveys (DHS), and other similar national HIV surveys (e.g., the South African National HIV Prevalence, Incidence and Behavior Survey) [12–22]. We will also search HIV conference abstracts including the International AIDS Society (IAS), the Conference on Retroviruses and Opportunistic Infections (CROI), and the International Conference on AIDS and STIs in Africa (ICASA) [23–25]. For eligible grey literature sources, we will review listed citations for additional data sources.

#### Search strategy

A detailed search strategy is presented in Additional file 2. In brief, MeSH and keywords will be included for HIV, such as “Human Immunodeficiency Virus” and “AIDS”, along with terms for each portion of the 90-90-90 cascade, and key terms for “Sub-Saharan Africa” and specific country names using Boolean “AND” and “OR” operators. For the first 90, we will include terms such as “testing”, “diagnosis”, and “serostatus”. The search for the second 90 will include terms such as “antiretroviral” and “treatment”, and the search for the third 90 will include terms such as “viral suppression” and “viral load”. Lastly, for our fourth search, we will include terms to capture the whole cascade, including “90-90-90”, “HIV care cascade”, and “universal test and treat”. Prior to publishing results, we will update our search to include any additional eligible papers that are published after March 2018.

### Study records

#### Data management

Each of the separate four searches will be conducted and merged into a reference manager. We will record the number of duplicate records, which will then be removed prior to the selection process. We will use Microsoft Excel and Covidence, a systematic review management software, to document the outcome of the title screening, abstract screening, full text review, and data abstraction processes [26].

#### Selection process

We will first screen records for inclusion based on title only, duplicated independently by two reviewers. In cases of disagreement, the record in question proceeds to the abstract review stage. Abstract review will also include duplicate reviews. Full texts of the records will then be obtained and reviewed for inclusion and will be conducted independently and in duplicate. Discrepancies from the abstract and full text review will be resolved through consensus or in discussion with a third independent reviewer. Records will be excluded from consideration at title, abstract, and full text review stages if they satisfy any of the following exclusion criteria: study only among HIV-negative persons, study on persons outside of sub-Saharan Africa setting, study on children aged under 15, study of an inappropriate design, study data collected exclusively prior to January 2014, or study does not report on at least one of the 90-90-90 outcomes (Table 2).

**Table 2:** Eligibility criteria

Criteria	Variables
Inclusion criteria	Study includes HIV-positive persons
	Study set in sub-Saharan Africa
	Study on adults aged 15 and older
	Study design is interventional, cohort, cross-sectional, case-control
	Study data collected at least partially from January 2014 onwards
	Study reports results on at least one 90-90-90 target
Exclusion criteria	Study includes only HIV-negative persons
	Study not set in sub-Saharan Africa
	Study includes children aged under 15
	Study design is qualitative, mathematical model, systematic review, or editorial
	Study data collected exclusively prior to January 2014
	Study does not report on any of the 90-90-90 targets

#### Data collection process

We will develop a data extraction form and pilot test this form on ten randomly selected publications which have been selected for data abstraction. This form will guide the collection of strata-specific estimates of inclusion across the three 90-90-90 targets, as well as strata definitions. Data extraction will be done independently by two reviewers, with discrepancies resolved by consensus or in consultation with a third reviewer.

#### Data items

The data items for extraction are informed by the study aims. We will include the following information from all studies:

- Study characteristics (authors, year of publication, study period, study design, geographic location, duration of follow-up, key findings)
- Study setting, specifically differentiating between clinical, community, and population-based study populations
- Definition and methods used to define the reported levels of the cascade, such as the threshold for virologic suppression
- Definitions and inclusion criteria used for specific subgroups defined by age, sex, occupation, migratory/mobile status, and risk behavior as well as key population definitions used
- For each population strata for which data are reported, the sample size, 95% confidence intervals, proportions, relative risk or odds ratio of PLHIV who know their HIV status (first 90), PLHIV who know their status and are on ART (second 90), and PLHIV on ART who are virologically suppressed (third 90)

#### Data synthesis

We will summarize information on virological suppression, and strata associated with being virologically suppressed. Strata reported, definitions used to define strata and levels of the HIV care cascade, and study characteristics including geography and study design will be summarized in the narrative. Study characteristics such as year and study period will be reported separately for each included study. Our qualitative assessment of the comparability of these definitions will inform our quantitative analysis.

We will summarize information on strata associated with knowledge of HIV serostatus, enrollment on ART, and virological suppression. This will include synthesizing odds ratios, relative risks, and proportions of persons virologically suppressed. We will hand calculate measures of association, odds ratio and risk ratio point estimates without confidence intervals, for studies which do not report or calculate measures of association but provide sufficient data for their calculation. Where appropriate and feasible, we will use random effects meta-regression analyses to estimate the distribution of demographic and risk features among groups of combinable strata who are not virologically suppressed. We hypothesize that national-level epidemic characteristics such as HIV prevalence and progress towards the 90-90-90 goals will be critical contextual features to consider in quantitative meta-analyses.

#### Missing or incomplete data

In the case of missing or incomplete data, we will contact corresponding authors to request relevant information or for additional clarification. If the corresponding authors fail to respond, then additional authors will be contacted. A description of the missing data for each included study will be provided, along with the possible implications of missing data. We will assess potential publication bias through the use of funnel plots and a subjective assessment of asymmetry [18].

#### Risk of bias in included studies

There will be three primary reviewers of included studies. At least two reviewers will assess the risk of selection bias, reporting bias, and attrition bias for each study included for data abstraction by using an adapted Cochrane Risk of Bias Tool including items for non-randomized studies [27]. Using this tool, reviewers will grade studies as having low, moderate, serious, or critical risk of bias. Conflicting ratings will be resolved through consultation of a third reviewer. We intend to assess the heterogeneity of study results by calculation of the *I*^2^ statistic [28]. Studies with critical risk of bias will not be considered for quantitative synthesis.

## Discussion

The effectiveness of the UNAIDS 90-90-90 targets to curb the HIV epidemic by 2030 may depend on reaching individuals with the highest risk of onward HIV transmission. In addition, successful achievement of these targets will mean that 27% of PLHIV are not virologically suppressed. Little is known about the demographic profile of those accessing the HIV care cascade and perhaps more importantly those people who are falling off the HIV care cascade. This systematic review and meta-analysis will help identify key demographic, epidemiologic, and risk factor heterogeneity across the 90-90-90, with a specific focus on correlates of being among the 27% who are not virologically suppressed. This will include those who fail to test and get linked to care, in addition to those on ART who do not achieve suppression. Results from this review will be useful in more accurately predicting the impact of achieving the 90-90-90 targets for reducing global HIV incidence and guiding health intervention and prevention strategies to more effectively reach key populations.

## Additional files

Additional file 1: PRISMA-P 2015 checklist. (125 kb)

Additional file 2: Search Terms. (19 kb)

## Abbreviations

AIDS: Acquired immune deficiency syndrome
AIS: AIDS Indicator Surveys
ALPHA: Analyzing Longitudinal Population-based HIV/AIDS data on Africa
ART: Antiretroviral treatment
CD4: Cluster of differentiation 4
CROI: The Conference on Retroviruses and Opportunistic Infections
DHS: Demographic and Health Surveys
HIV: Human immunodeficiency virus
IAS: The International AIDS Society
IBBS: Integrated Bio-behavioral Surveys
ICASA: The International Conference on AIDS and STIs in Africa
MSF: Médecins Sans Frontières
NTRL: National Technical Reports Library
PEPFAR: U.S. President’s Plan for AIDS Relief
PHIA: Population-based HIV Impact Assessments
PLHIV: People living with HIV
PRISMA-P: Preferred Reporting Items for Systematic Review and Meta-Analysis Protocols
PROSPERO: International Prospective Register of Systematic Reviews database
UNAIDS: Joint United Nations Programme on HIV/AIDS
UTT: Universal test and treat
WHO: World Health Organization
